# Hyperfractionated abdominal reirradiation for gastrointestinal malignancies

**DOI:** 10.1186/s13014-018-1084-0

**Published:** 2018-08-07

**Authors:** Andrew Hunt, Prajnan Das, Bruce D. Minsky, Eugene J. Koay, Sunil Krishnan, Joseph M. Herman, Cullen Taniguchi, Albert Koong, Grace L. Smith, Emma B. Holliday

**Affiliations:** 1 University of Texas Health San Antonio Long School of Medicine, San Antonio, TX USA; 2 The University of Texas MD Anderson Cancer Center, 1515 Holcombe Blvd Unit 97, Houston, TX 77030 USA

**Keywords:** Reirradiation, External beam, Hyperfractionated, Abdominal, Gastrointestinal malignancies, Toxicity, Local control

## Abstract

**Background:**

We sought to determine the role of abdominal reirradiation for patients presenting with recurrent or new primary gastrointestinal (GI) malignancies. At our institution, we have established a hyperfractionated, accelerated reirradiation regimen consisting of 39 Gray (Gy) in 26 twice-daily fractions. Although this regimen is used frequently in the pelvis, we sought to determine its toxicity and efficacy for abdominal tumors.

**Methods:**

Twenty-four patients who received abdominal reirradiation with a hyperfractionated, accelerated approach from 2000 to 2017 were identified. Overall survival (OS) and local progression-free survival (LPFS) were calculated using the Kaplan-Meier method. Several patient, tumor and treatment characteristics were evaluated on univariate analyses for association with OS and LPFS using a Cox proportional hazards model.

**Results:**

Of the twenty-four patients identified, the majority (*n* = 11, 46%) had pancreatic adenocarcinoma as their primary disease but also included upper GI adenocarcinoma (*n* = 4), colon adenocarcinoma (*n* = 3), hepatobiliary cancers (*n* = 4) and other malignancies (*n* = 2). The majority of patients received 45–50.4Gy in 1.8Gy fractions as their initial abdominal radiation course. The median reirradiation dose was 39Gy in 26 twice-daily fractions with a minimum six hour interval. The median [interquartile range (IQR)] interval between the courses of radiotherapy was 28 [18.6–38.9] months. Only palliative reirradiation intent was associated with decreased OS. While colon adenocarcinoma primary was significantly associated with increased LPFS, the sample size was small (*n* = 3). The 1-yr rate of LPFS was 38%. The median [IQR] duration of freedom from local progression was 8 [3.8–19.2] months. The 1-year OS was 50% and the median (IQR) OS was 14 [6.3–19.6] months. Thirteen patients (54%) had acute side effects with one patient experiencing G3 nausea and one experiencing a G4 bleed; the remaining patients experienced G1-G2 symptoms.

**Conclusion:**

Hyperfractionated, accelerated reirradiation to the abdomen was relatively well-tolerated but provided limited local control to recurrent or second primary abdominal malignancies. Reirradiation could play a role in treating these patients with palliative or curative intent, but alternative strategies for delivering increased biologically effective dose should be further explored.

## Background

Given the recent advances in systemic therapy for several gastrointestinal malignancies, local recurrences (LR) can be life- and/or quality-of-life-limiting. For patients with pancreatic adenocarcinoma, LR occurs in 80% of patients within two years of curative-intent therapy, and 30% of these LR are isolated without distant metastasis (DM) [1–3]. For patients with gastric cancer, LR rates are closer to 33%, although the majority of these patients also present with synchronous distant metastatic disease [4]. Younger patients and patients with larger tumor sizes are at highest risk for LR [5]. For patients with colon adenocarcinoma, LR rates are also approximately 32% following curative therapy [6].

The treatment of locally recurrent tumors in the abdomen varies depending on the specific histology, location and extend of recurrence as well as any prior treatments received. For pancreatic cancer recurring after resection, re-resection is favored if feasible, but radiation-based modalities are not recommended for patients who have received prior (chemo) radiation therapy [7]. Re-resection is also favored for locally recurrent gastric, colon and hepatobiliary cancers when feasible [8–10]. The need for alternative treatment strategies arises as patients are often not surgical candidates at the time of local recurrence either because of technical operability or medical comorbidity. Additionally, effective palliation of symptomatic LR occurring in patients with concomitant regional or metastatic disease. Radiation therapy is one such alternative local modality, but its utilization in the treatment of recurrent abdominal tumors has been limited by the increasing use of primary radiation in the neoadjuvant or adjuvant settings for abdominal tumors in the pancreas, stomach, colon and hepatobiliary region.

Previous studies have established the safety and efficacy of reirradiation to the head and neck, pelvis, breast, brain, breast, and lung [11–25]. However, reirradiation is not commonly used in patients with recurrent GI malignancies due to concerns of toxicity, with a cumulative radiation dose that exceeds established dose constraints. At our institution, a hyperfractionated accelerated reirradiation approach has been shown to be effective in the treatment of both recurrent rectal cancer and anal cancer [26–28]. Patients treated with reirradiation for recurrent rectal cancer using 1.5Gy twice daily fractions to a total median dose of 39Gy demonstrated a 3-year freedom from local progression (FFLP) rate of 40% and a 3-year overall survival (OS) rate of 39% [27]. Patients who underwent reirradiation in twice 1.5Gy daily fractions for recurrent anal cancer demonstrated a 3-year FFLP of 56% and a 3-year OS of 60% [28].

Haque et al. first demonstrated the safety and efficacy of abdominal reirradiation in patients with gastrointestinal malignancies, showing a 1-yr rate of freedom from local progression of 50% and a 1-yr rate of overall survival of 62% [29]. This initial report, however, was small (*n* = 13) [29]. Our aim with the current study was to update this initial experience and report the efficacy and toxicity associated with this hyperfractionated, accelerated reirradiation approach to recurrent or new primary tumors in the abdomen.

## Methods

Between January 1 2000 and January 1 2017, 24 patients with gastrointestinal cancer and a prior history of radiation therapy had undergone reirradiation using a hyperfractionated, accelerated approach. The hospital records for these treatments were reviewed with the approval of the University of Texas MD Anderson Cancer Center Institutional Review Board.

### Reirradiation details

Prior radiotherapy records were obtained and reviewed by the treating radiation oncologist. When available, prior radiotherapy records in Digital Imaging and Communications in Medicine (DICOM) format were obtained and a composite plan was generated with both the original and reirradiation dose-distributions, target coverage and dose to adjacent organs-at-risk (OARs).

For the purposes of planning reirradiation, all patients underwent a computed tomography (CT) simulation. Intravenous contrast was used at the discretion of the treating physician and was used more often in cases of hepatobiliary tumor location. Patients were asked to fast for three hours prior to simulation and treatment for stomach volume reproducibility. Abdominal compression was not used. Gross tumor volume (GTV), defined as the primary tumor and any malignant-appearing lymphadenopathy, was delineated by the treating physician based on all available diagnostic imaging. The clinical target volume (CTV) typically included the GTV plus a 1–2 cm margin. Elective nodal coverage was not included. The planning target volume (PTV) typically included a 5 mm margin for set up uncertainties with the use of daily kilovoltage image-guidance. Respiratory motion uncertainty management with either a 4DCT scan or the use of a breath hold technique was considered depending on tumor location and proximity to critical OARs. Either 3D conformal radiation therapy (3DCRT) or intensity-modulated radiation therapy (IMRT) was utilized at the discretion of the treating physician.

### Assessment of cancer control and toxicity endpoints

Patients were assessed by their treating radiation oncologist at least every five fractions (twice weekly). Radiation-related toxicities were described and graded according to the Common Toxicity Criteria for Adverse Events (CTCAE), version 4.0 [30]. Acute toxicities were defined as those occurring between the initiation of radiation and six weeks after the last radiation treatment. Late toxicities were defined as those occurring and/or persisting more than 6 weeks after the last radiation treatment. All of the data collected and analyzed are retrospective in nature. Follow up was every 1–4 months for the first two years after treatment as clinically indicated at the discretion of the treating team. Dates and locations of progressive disease were recorded as well as vital status at last follow-up. Follow up information was obtained from hospital records and radiation therapy records. Follow-up information was also obtained from the MD Anderson Tumor Registry, which collects information on patients annually through letters and phone calls.

### Calculation of composite maximum doses for luminal gastrointestinal organs

For patients for whom initial radiation records and composite plans were available, the maximum point dose from the initial course, the dose from the reirradiation course and the composite dose to the stomach, duodenum, small intestine and large intestine were collected. Next, the equivalent dose in fractions for the initial course, the reirradiation course and the composite plans were calculated for each patient using the following equation:

$$ {EQD}_{\mathrm{r}}^{\mathrm{f}}=D\left(\frac{d+r}{f+r}\right) $$

Where D is the total dose in Gy given in d Gy fractions of d = 2 Gy each using an r α/β ratio of 4. As published reports of late bowel toxicity have shown the appropriate α/β ratio is 3–5 [31–34].

### Statistical analysis

Chi-square test was used for between-group comparisons of categorical variables, and the Mann-Whitney U test was used for between-group comparisons of continuous variables. *P*-values <.05 were considered significant. Local progression-free survival (LPFS) was calculated from the initiation of reirradiation to the date of local disease progression, death from any cause or last follow-up. Overall survival (OS) was calculated from the initiation of reirradiation to the date of death from any cause or last follow-up. Analysis of LPFS and OS was performed by the Kaplan-Meier method [35]. Cox’s proportional hazards model was used for univariate and multivariate analyses to evaluate potential prognostic factors for LPFS and OS. The hazard ratio (HR) is reported with the 95% confidence interval (CI) for each variable. Factors with a *p*-value <.2 on univariate analysis were included in the multivariate model. The statistical software used was JMP version 12 (SAS Institute Inc., Cary, NC).

## Results

Twenty-four patients were included in our analysis with a median [interquartile range (IQR)] follow up of 16.8 [8–22.5] months.

### Patient characteristics

Patient characteristics are shown in Table 1. The median [IQR] age at the time of retreatment was 65 [54.1–69.1] years. The primary cancer diagnoses were as follows: eleven patients with pancreatic adenocarcinoma, four patients with upper GI adenocarcinoma, three patients with colon adenocarcinoma, four patients with hepatobiliary cancers, and two patients with other malignancies. The majority of patients received standard fractionated radiation (1.8Gy per day) to a total dose of 45–50.4Gy, but one patient received 30Gy/24 fractions, four patients received 30Gy/10 fractions, one patient received 30Gy/12 fractions and two patients received 35Gy/14 fractions. Twenty patients (83%) were treated with 3DCRT and 4 patients (17%) were treated with IMRT. The median [IQR] interval between the two treatments of radiation was 27.9 [18.6–38.9] months. Twenty-one patients (87.5%) received concurrent chemotherapy with their first course of radiation.

**Table 1:** Patient and Treatment Characteristics

Characteristic	Median [IQR] or Number (%)
Age at 2nd Radiation Treatment (years)	65 [54.1–69.1]
Sex	
Male	16 (67%)
Female	8 (33%)
Pathology of Initial Primary	
Pancreatic Adenocarcinoma	11 (46%)
Upper GI Adenocarcinoma^a^	4 (16.7%)
Colon Adenocarcinoma	3 (12.5%)
Hepatobiliary Cancers^b^	4 (16.7%)
Other^c^	2 (8.3%)
Dose/fractionation of 1st Radiation Treatment (total dose in Gray (Gy)/total number of fractions)	
30Gy/24 fractions	1 (4.2%)
30Gy/10 fractions	4 (16.7%)
30Gy/12 fractions	1 (4.2%)
35Gy/14 fractions	2 (8.3%)
45Gy/25 fractions	8 (33.3%)
50.4Gy/28 fractions	8 (33.3%)
Retreatment Interval (months)	27.9 [18.6–38.9]
Type of Post-1st Radiation Disease	
Recurrent Disease	21 (87.5%)
Second Primary Tumor	3 (12.5%)
Location of Post-1st Radiation Disease	
Pancreas	14 (58.3%)
Stomach/Duodenum	5 (20.8%)
Liver	2 (8.3%)
Other^d^	3 (12.5%)
Reirradiation Intent	
Definitive/Local Control	16 (67%)
Palliation of Bleeding	5 (20.8%)
Palliation of Pain	2 (8.3%)
Palliation of Tumor Thrombus	1 (4.2%)
Reirradiation Dose^e^ (total dose in Gray (Gy)/total number of fractions)	
15Gy/10 fractions	1 (4.2%)
30Gy/20 fractions	7 (29.2%)
39Gy/26 fractions	15 (58.3%)
45Gy/30 fractions	1 (4.2%)
Reirradiation Technique	
3D Conformal	15 (62.5%)
Intensity-modulated radiation therapy	9 (37.5%)
Concurrent Chemotherapy	
Yes	17 (70.9%)
No	7 (29.2%)

*IQR* interquartile range

^a^Upper GI Adenocarcinoma = adenocarcinomas of the gastroesophageal junction (*N* = 1), stomach (*N* = 1) and duodenum (*N* = 2)

^b^Hepatobiliary Cancers = intrahepatic cholangiocarcinoma (*N* = 2), extrahepatic cholangiocarcinoma (*N* = 1) and hepatocellular carcinoma (*N* = 1)

^c^Other = pancreatic neuroendocrine tumor (N = 1), Hodgkin Lymphoma treated with mantle and abdominal fields (*N* = 1)

^d^Other = Mesenteric adenopathy, peritoneal nodules, aortocaval adenopathy

^e^Reirradiation was given in twice daily fractions with a minimum 6 h interfraction interval

At the time of reirradiation, three patients (12.5%) had new primary tumors while 21 patients (87.5%) had recurrent disease. All of the patients were evaluated by a surgeon at our institution and were deemed inoperable either because they had distant metastatic disease (*N* = 3), they had comorbidities that precluded surgery (*N* = 2) or they were anatomically unresectable given the location of their tumor (*N* = 19). The median [IQR] length of time between the two courses of radiation was 28 [18.6–38.9] months. The site of reirradiation was as follows: 14 patients were treated to the pancreas, five to the stomach/duodenum, two to the liver, one to mesenteric adenopathy, one to peritoneal nodules, and one to aortocaval adenopathy. Sixteen patients (66.7%) were treated with definitive intent for local control, while eight patients (33.3%) were treated for the palliation of tumor-related symptoms. Twelve patients (50%) received chemotherapy prior to receiving their second radiation treatment.

All patients received accelerated, hyperfractionated reirradiation with 1.5Gy delivered per fraction and two fractions delivered per day with a minimum 6 h interfraction interval. Seven patients received 30Gy/20 fractions, fifteen received 39Gy/26 fractions, one received 45Gy/30 fractions and one patient stopped treatment early after 15Gy in 10 fractions. Fifteen patients (62.5%) received 3DCRT with the remaining nine patients (37.5%) receiving IMRT. Seventeen patients (70.4%) received concurrent chemotherapy, with 14 patients receiving capecitabine, one patient receiving capecitaibine and erlotinib, one patient receiving gemcitabine and erlotinib, and one patient receiving sorafenib. The mean ± SD gross tumor volume for patients in this series was 105.4 ± 119.4ccs, and the median [IQR] was 59ccs [27.8-140ccs]. Three patients had involved nodes treated.

### Patient outcomes

**Fig. 1:**
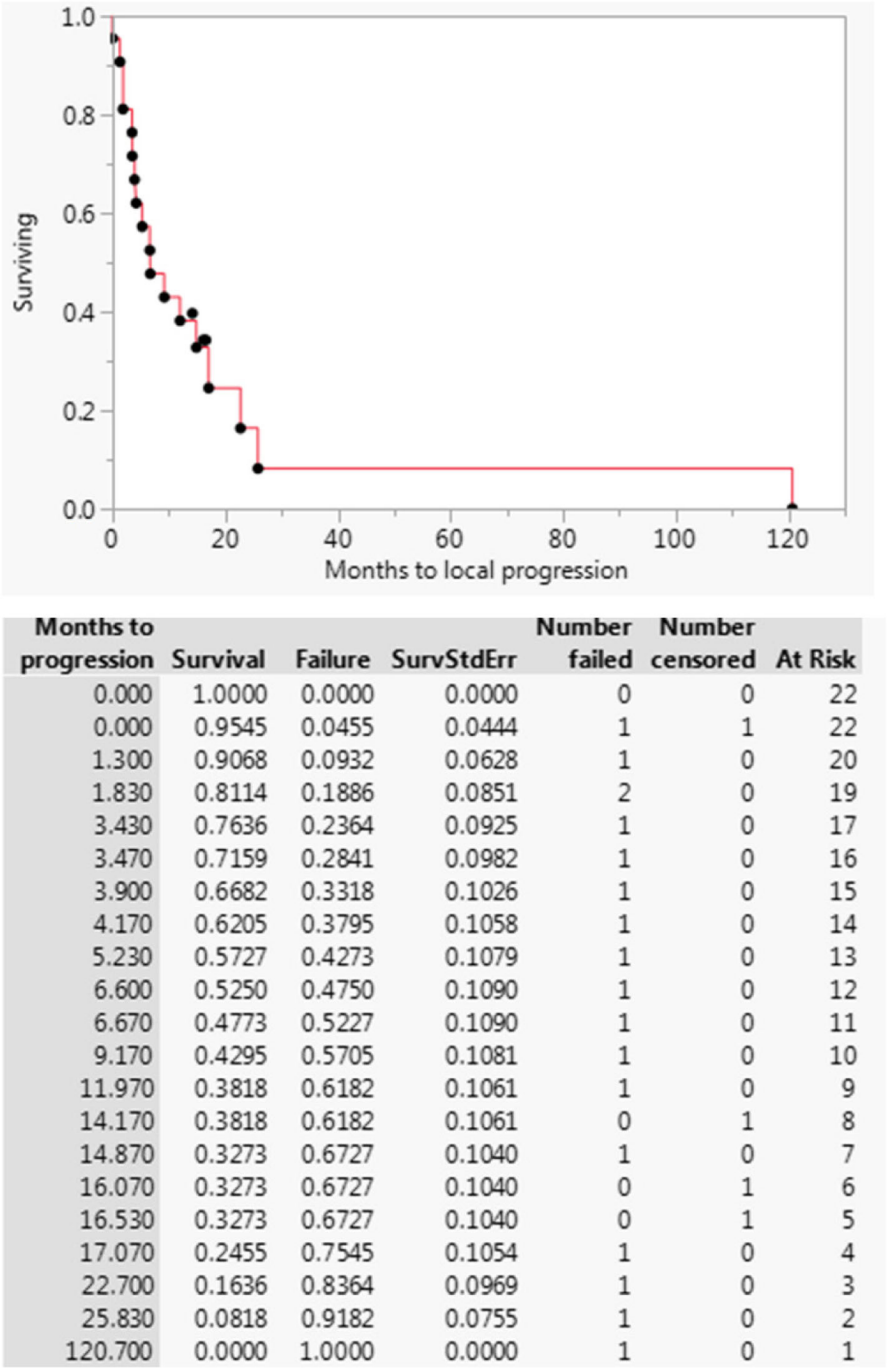
Local progression-free survival for patients undergoing hyperfractionated accelerated reirradiation for abdominal malignancies

**Fig. 2:**
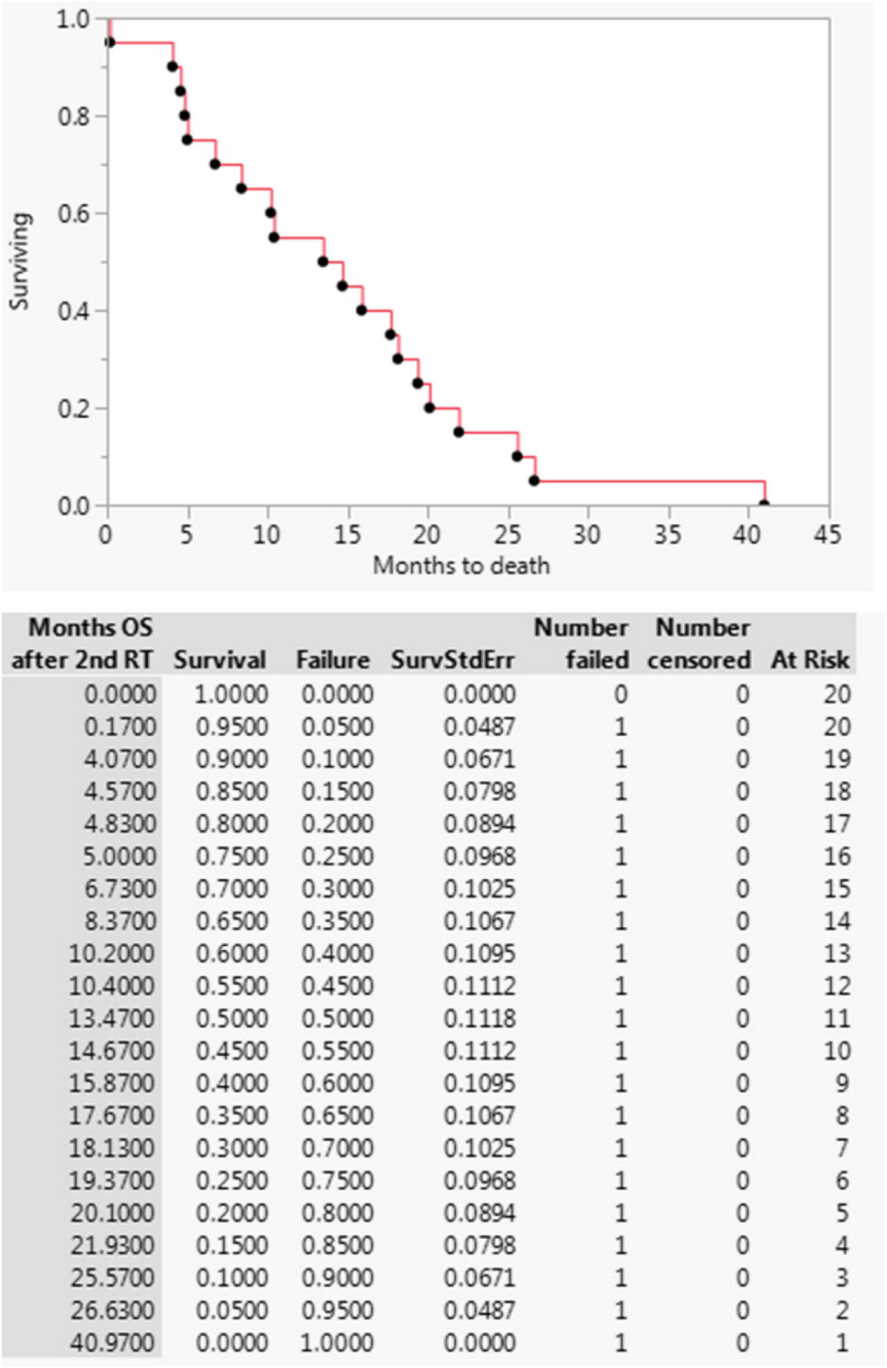
Overall survival for patients undergoing hyperfractionated accelerated reirradiation for abdominal malignancies

The 1-year LPFS was 38%, and the median [IQR] duration of freedom from local progression was 8 [3.8–19.2] months (Fig. 1). Results of univariate analyses performed for LPFS and OS are given in Table 2. Only colon adenocarcinoma primary type was significantly associated with increased LPFS, but the sample size was small (*n* = 3), with two patients not progressing and a third patient not progressing for 10 years. The 1-year OS was 50% and the median [IQR] overall survival was 14 [6.3–19.6] months (Fig. 2). Only palliative reirradiation intent was significantly associated with decreased OS (HR 3.38 95% CI 1.24–9.03; *p* = .018).

**Table 2:** Univariate analyses of factor associated with local progression-free survival and overall survival for patients receiving abdominal re-irradiation using a hyperfractionated regimen

	Local progression-free survival			Overall survival		
Factor	Hazard ratio	95% Confidence interval	*P*-value	Hazard ratio	95% Confidence interval	*P*-value
Age at Reirradiation						
< 65	Reference	Reference		Reference	Reference	
≥ 65	0.72	0.27–1.89	.501	0.60	0.24–1.48	.268
Gender						
Male	Reference	Reference		Reference	Reference	
Female	0.87	0.27–2.54	.806	0.58	0.20–1.46	.256
Primary Cancer						
Pancreatic Adeno	Reference	Reference		Reference	Reference	
Upper GI Adeno^a^	1.14	0.24–4.07	.856	1.36	0.20–4.60	.650
Colon Adeno	*1.12 × 10* ^–9*^	*5.67 × 10* ^*− 55*^ *-.35* ^*^	*.002* ^*^	3.65	0.76–13.82	.098
Hepatobiliary^b^	0.78	0.17-2.72	.715	1.02	0.23–3.44	.976
Other^c^	0.66	0.03-3.66	.678	2.34	0.34–9.95	.334
Initial RT dose						
≤ 45Gy	Reference	Reference		Reference	Reference	
> 45Gy	0.62	0.20–1.67	.361	1.09	0.41–2.69	.848
Interval between 1st and 2nd courses of RT						
< 1 year	Reference	Reference		Reference	Reference	
≥ 1 year	1.19	0.12–3.28	.824	0.45	0.14–2.00	.261
Type of post-1st RT disease						
New Primary	Reference	Reference		Reference	Reference	
Recurrence	2.37	0.46–43.24	.351	0.50	0.16–2.22	.323
Reason for reirradiation						
Definitive	Reference	Reference		Reference	Reference	
Palliative	1.42	0.48–3.80	.510	3.38	1.24–9.03	.018
Reirradiation dose						
≤ 30Gy	Reference	Reference		Reference	Reference	
> 30Gy	1.31	0.43–4.96	.650	1.10	0.44–3.13	.844
Reirradiation modality						
3DCRT	Reference	Reference		Reference	Reference	
IMRT	0.62	0.21–1.64	.342	0.84	0.31–2.06	.708
Concurrent chemo with reirradiation?						
No	Reference	Reference		Reference	Reference	
Yes	1.51	0.51–5.55	.473	0.75	0.30–2.13	.565

*Only colon adenocarcinoma primary type was significantly associated with increased LPFS, but the sample size was small (*n* = 3), with two patients not progressing and a third patient not progressing for 10 years

^a^Upper GI Adenocarcinoma = adenocarcinomas of the gastroesophageal junction (*N* = 1), stomach (*N* = 1) and duodenum (*N* = 2)

^b^Hepatobiliary Cancers = intrahepatic cholangiocarcinoma (*N* = 2), extrahepatic cholangiocarcinoma (*N* = 1) and hepatocellular carcinoma (*N* = 1)

^c^Other = pancreatic neuroendocrine tumor (*N* = 1), Hodgkin Lymphoma treated with mantle and abdominal fields (*N* = 1)

Eight patients (33.3%) were treated with palliative intent, five for palliation of bleeding, two for palliation of pain, and one for palliation of a tumor thrombus. Of these, only two patients (25%) experienced noticeable palliation of the target symptoms.

### Toxicity

Toxicity details are given in Table 3. Thirteen patients (54%) experienced acute side effects, although most were mild and consisted of G1-G2 symptoms including nausea, dyspepsia, diarrhea and esophagitis. One patient experienced G3 nausea and one experienced an acute G3 bleed due to an ulcer in the radiation field. The patient who experienced bleeding from the gastrojejunal anastomosis required a 4 day hospitalization and subsequent termination of the remaining scheduled radiation treatments. At the time radiation treatments were terminated, the patient had received 30 of a planned 39Gy, and the cumulative dose to the gastrojejunal anastomosis was 63Gy. This patient subsequently developed further bleeding from the gastrojejunal anastomosis qualifying as a grade 4 chronic toxicity two months after completion of reirradiation. The patient required endoscopic clips, epinephrine and coagulation and ultimate surgical revision of the gastrojejunostomy. One additional patient had their radiation treatment stopped early (also at 30 of a planned 39Gy) due to the development of a grade 2 duodenal ulcer which did not progress further. One additional patient developed severe GI symptoms found to be due to progression of peritoneal disease after they received 15 of a planned 39Gy. After discussion with the patient, their family and the multidisciplinary team, the remaining radiation treatments were cancelled and the patient went on to receive hospice care. All remaining patients completed the prescribed reirradiation course.

**Table 3:** Acute Toxicities

Characteristic	Number (%)
Patients Experiencing Acute Side Effects During Reirradiation	13 (54%)
Acute RT Side Effects^a^	
G1–2 Nausea	10 (41.7%)
G3 Nausea	1 (4.2%)
G1–2 Diarrhea	1 (4.2%)
Other G1–2 side effects	3 (12.5%)
G4 GI bleed	1 (4.2%)
Patients Experiencing Acute 2nd RT Side Effects	2 (8.3%)
Acute RT Side Effects	
G3 Nausea	1 (4.2%)
G3 Pancreatitis and Biliary Sepsis	1 (4.2%)

^a^Some patients experienced more than one toxicity

### Cumulative doses to luminal gastrointestinal organs

**Fig. 3:**
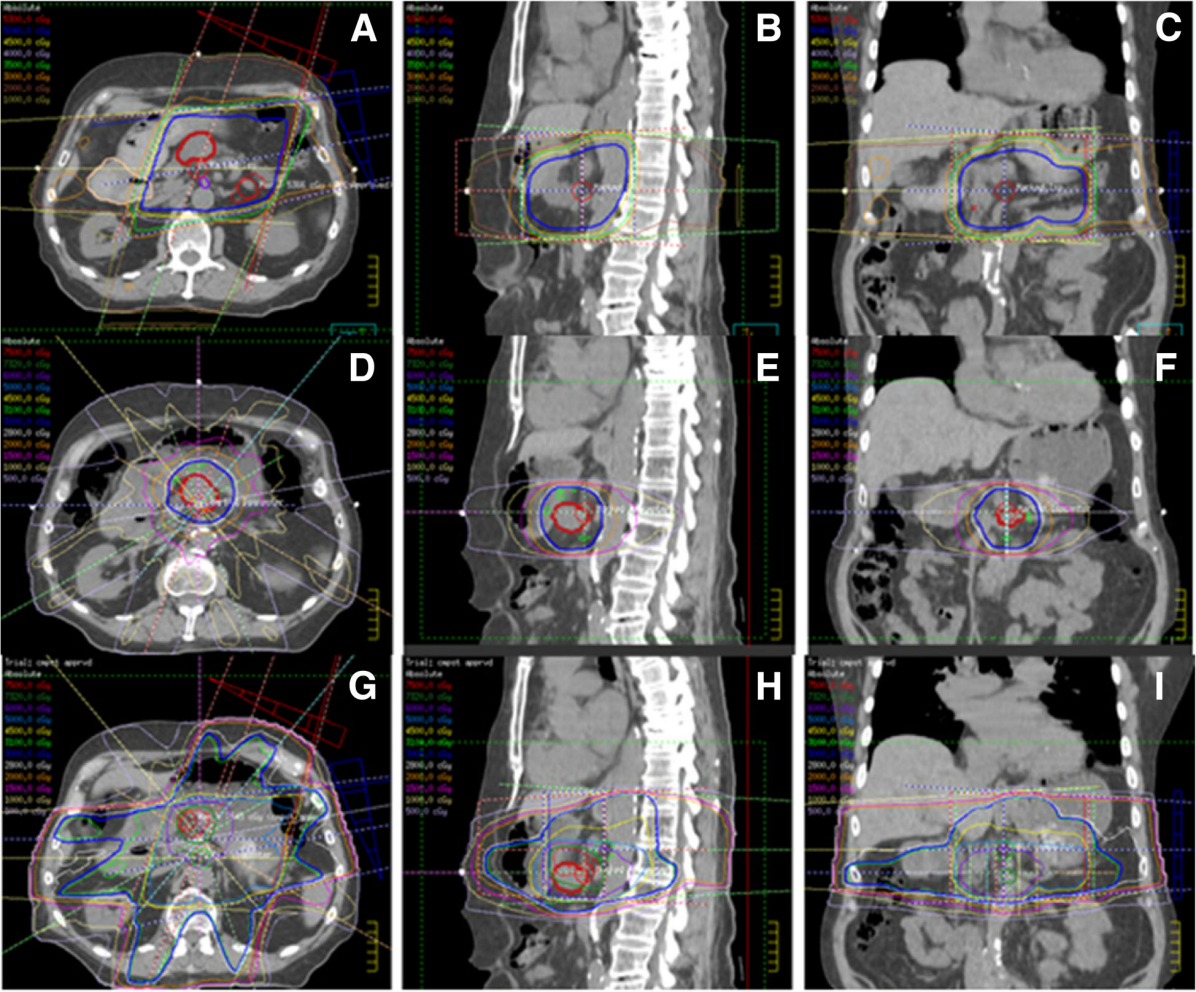
A representative patient with pancreatic adenocarcinoma who was initially planned to receive 50.4Gy in 28 fractions using a 4-field 3D conformal radiation plan (**a**-**c**). Unfortunately, he only received 43.2Gy in 24 fractions before having to stop capecitabine-based chemoradiation due to a family emergency. He was subsequently unable to undergo surgical resection due to anatomic unresectability. Thirteen months later, he developed isolated local progression and was treated to a total dose of 30Gy in 20, twice-daily fractions of 1.5Gy each using an intensity-modulated radiation therapy technique (**d**-**f**). He received concurrent oral capecitabine chemotherapy. The cumulate plan is shown (**g**-**i**)

Detailed dosimetric data sufficient for creating a composite radiation plan were available for 14 (58.3%) patients in our cohort. For these patients, maximum doses to luminal gastrointestinal organs from the original, reirradiation and composite plans are given in Table 4. Original, reirradiation and composite doses are given in both maximum nominal dose as well as maximum equivalent dose in 2 Gy fractions with an alpha beta ratio of 4 (EQD_2_^4^). No significant relationships were found between maximum GI organ dose and G3+ toxicity. Figure 3 shows the original plan, the reirradiation plan and the composite plan for the treatment of locally recurrent pancreatic adenocarcinoma.

**Table 4:** Initial Radiation, Reirradiation and Cumulative Maximum Dose to Bowel

Location	Initial treatment dose in gray median [range] mean ± SD		Reirradiation treatment dose in gray median [range] mean ± SD		Cumulative dose in gray median [range] mean ± SD	
	Nominal dose	EQD_2_^4a^	Nominal dose	EQD_2_^4a^	Nominal dose	EQD_2_^4a^
Stomach	34.6 [0–56.1] 35.9 ± 15.1	36.9 [0–51.8] 35.7 ± 14.5	34.6 [1.4–48] 30.0 ± 14.2	31.7 [0.94–44.8] 27.4 ± 13.5	68.8 [32–92.2] 65.9 ± 15.3	66 [29.9–88.1] 63.1 ± 15.2
Duodenum	40.5 [21–55.7] 39.9 ± 10.2	43.9 [16.3–55.5] 41.2 ± 10.2	39.2 [0–48] 35.6 ± 11.3	35.7 [0–44.8] 32.9 ± 10.5	75.7 [50.4–95.4] 75.5 ± 13.0	76.3 [46.0–89.8] 74.1 ± 13.1
Small Bowel	39.6 [20.7–52.8] 37.9 ± 10.7	42.2 [16.2–52.3] 38.6 ± 11.5	35.6 [0–48] 32.9 ± 12.8	31.9 [0–44.8] 30.2 ± 12.3	71.4 [50.4–95.4] 70.8 ± 12.9	67.9 [46.1–89.8] 68.7 ± 14.0
Large Bowel	36.1 [20.9–52.8] 37.0 ± 11.3	38.4 [16.3–51.7] 37.2 ± 11.6	35.9 [0–48.1] 32.5 ± 13.2	32.1 [0–44.8] 29.8 ± 12.5	68.1 [50.4–98.1] 69.5 ± 13.5	66.4 [46.1–93.1] 67.0 ± 14.0

^a^EQD_2_^4^ is the equivalent dose in two Gray fractions using an alpha beta ratio of 4 for late toxicity effects on the luminal abdominal GI organs

## Discussion

The results of this retrospective review performed at a high-volume tertiary referral cancer center show that hyperfractionated, accelerated reirradiation can be safely delivered with acceptable toxicity rates for patients with recurrent or new primary abdominal tumors who have received prior abdominal radiation. These data expand upon our previously reported experience of the initial 13 patients treated was published in 2009. While abdominal reirradiation using this regimen was well tolerated, it appeared to have limited efficacy in terms of freedom from local progression and palliation of local symptoms.

In this cohort of patients, the 1-year LPFS was only 38%, and the median [IQR] time to local progression was only 8 [3.8–19.2] months. Patients with colon adenocarcinoma seemed to have a longer LPFS; two patients never experienced local progression and a third patient not progressing until 10 years after reirradiation. However, this group of patients was small (*n* = 3), so it would not be prudent to draw definitive conclusions from these results. Patients in this cohort had a poor prognosis overall with a 1-year OS of 50% and a median [IQR] overall survival of 14 [6.3–19.6] months. Patients treated with palliative intent had, predictably, a significantly worse overall survival, but this hyperfractionated, accelerated reirradiation regimen did not prove to be very effective in palliating symptoms of pain, bleeding or tumor thrombus. Only 25% of patients treated palliative reported noticeable symptom relief.

These modest results suggest alternative regimens should be explored to provide patients with better radiation therapy options in the abdominal reirradiation setting. Although the hyperfractionated schedule has the potential radiobiologic benefit of reducing the risks of late toxicities from cumulative radiation doses [36], particularly to sensitive luminal gastrointestinal structures, some groups have reported encouraging reports with a hypofractionated stereotactic body radiation therapy (SBRT) approach. The majority of published data come from studies reporting on reirradiation for pancreatic cancer using hypofractioned SBRT. Lomiska et al. utilized a regimen consisting of a median dose of 22.5Gy in 3 fractions to recurrent tumors of the pancreas and reported a median overall survival of only 5.9 months, but freedom from local progression was excellent at 85.7% (12 of 14 evaluable patients). Grade 3 or greater acute toxicities were reported in fewer than 10% of patients [37]. Dagoglu et al. reported a median OS of 14 months, two year local control of 78% and G3 of greater acute toxicity rate of 10% in patients treated for unresectable locally recurrent pancreatic adenocarcinoma using a median of 25 Gy in a median of 5 fractions [38]. Wild et al. reported a median survival of 8.8 months in pancreatic adenocarcinoma patients reirradiated with SBRT using the same median SBRT dose of 25Gy in 5 fractions. Rates of freedom from local progression at 6 and 12 months after SBRT reirradiation were 78 and 62%, respectively. Effective symptom palliation (back or abdominal pain) was higher than in our series at 57%. Only 28% of patients experienced acute toxicities, but no patient experienced G3 or greater acute toxicity. Analysis of this cohort also suggested that a progression-free interval of > 9 months prior to isolated local recurrent or progression may predict for a greater benefit of SBRT reirradiation as their survival is more likely to be long enough for improved local control to be of meaningful benefit [39]. Most recently, Koong et al. showed that either single-fraction (25Gy × 1) or five-fraction (5–6.6Gy × 5) SBRT was effective in treating recurrent pancreatic cancer a median of 13 months after patients had received prior conventional radiotherapy. The median overall survival from reirradiation was 8.5 months, and the local failure rate was low at 19%. Distant progression was a much more common pattern of failure at 64%. Reirradiation was also well tolerated with this regimen, with only six (26%) of patients experiencing either grade 2 or grade 3 toxicities, and four of these patients were treated with the single fraction SBRT regimen [40].

While our study demonstrated comparable overall survival rates, the prior studies using SBRT for reirradiation of pancreatic adenocarcinoma reported superior freedom from local progression. While our study included patients with gastrointestinal malignancies, the majority (*n* = 14, 58.3%) of patients included in this study were treated for pancreatic adenocarcinoma, and we would thus expect our one year freedom from local progression to be comparable if not higher. Furthermore, although our serious (G3 or greater) toxicity rate was low, our study reported an overall acute toxicity rate higher than those reported using SBRT, with a 54% acute toxicity rate in our study compared to 10 to 28% reported by the SBRT studies [37–40].

While this series represents the largest reported cohort of patients treated with hyperfractionated, accelerated abdominal reirradiation for recurrent or new primary abdominal malignancies, this study has several limitations that may limit its interpretation and broad applicability. The number of patients was relatively small, with only 24 patients treated over a 17 year period at a single institution. The cohort was relatively heterogeneous in terms of primary tumor type and prior treatment delivered. Additionally, acute toxicities were collected retrospectively based on hospital records and could have been underestimated. Follow up was not standardized, varied between patients, and late toxicities may have been underreported as well.

## Conclusion

In conclusion, although our study shows that hyperfractionated, accelerated abdominal reirradiation is well-tolerated, this regimen provides local control for a limited duration and has a modest palliative effect. Emerging data regarding SBRT reirradiation in the abdomen are promising, and may provide improved local control with similarly low toxicity rates. Further study is needed regarding the safety and efficacy of this modality of recurrent pancreatic adenocarcinoma as well as other abdominal malignancies.

## Abbreviations

3DCRT: Either 3D conformal radiation therapy
CI: Confidence interval
CT: Computed tomography
CTCAE: Common Toxicity Criteria for Adverse Events
CTV: The clinical target volume
DM: Distant metastasis
FFLP: Freedom from local progression
GI: Gastrointestinal
GTV: Gross tumor volume
Gy: Gray
HR: Hazard ratio
IMRT: Intensity-modulated radiation therapy
IQR: Interquartile range
LPFS: Local progression-free survival
LR: Local recurrences
OARs: Organs-at-risk
OS: Overall survival
PTV: The planning target volume
